# Editorial: Vascular neurosurgery and microneuroanatomy

**DOI:** 10.3389/fsurg.2023.1229789

**Published:** 2023-06-26

**Authors:** Feres Chaddad-Neto, Ricardo Silva Centeno, Marcos Devanir Silva da Costa, Kaan Yagmurlu, Juan Carlos Ahumada-Vizcaino, Raphael Wuo-Silva

**Affiliations:** ^1^ Department of Neurology and Neurosurgery, Universidade Federal de São Paulo, São Paulo, SP, Brazil; ^2^ Department of Neurosurgery, Hospital Beneficência Portuguesa de São Paulo, São Paulo, SP, Brazil; ^3^ Departments of Neurological Surgery and Neuroscience, University of Virginia School of Medicine, Charlottesville, VA, United States

**Keywords:** cerebrovascular diseases, microanatomy, vascular neurosurgery, neuroanatomy, clinical research

**Editorial on the Research Topic** Vascular neurosurgery and microneuroanatomy

This Research Topic entitled *Vascular Neurosurgery and Microneuroanatomy* aims to bring together advanced techniques for the treatment of diseases such as aneurysms, cerebral arteriovenous malformations, cavernomas, epilepsy, and tumors that affect the nervous system, combined with advanced neuroanatomical knowledge that can contribute to a better understanding of these diseases and ultimately a better patient outcome.

This Research Topic consists of six original articles, three case reports, one review, and one mini-review.

The first original paper by Du et al. focused on verifying whether stereotaxic aspiration could bring benefits to patients with brainstem hemorrhage. Two groups of patients with brainstem hemorrhage received either stereotaxic aspiration (*n* = 42) or conservative treatment (*n* = 30). To assess these groups, the authors used CT scan images and Glasgow Scale Coma (GSC) and Modified Rankin Scale (mRS) score. They concluded that stereotaxic aspiration is effective in evacuating brainstem hemorrhage and promoting rapid recovery and better clinical outcomes for patients.

The second original paper by Hu et al. performed a retrospective study including three patients with blood blister-like aneurysm and two patients with communicating artery aneurysm to evaluate the effectiveness of the clip-reinforced wrapping technique with the Y-shaped temporalis fascia (CRYST). Overall, this study showed that the CRYST technique had favorable results in patients with intractable intracranial aneurysms, without neurological deficits after surgery. In addition, at follow-up, the patients did not present aneurysm or stenosis of the parent artery in the angiography performed 10–14 days after surgery.

The third original paper by Sun et al. performed a retrospective study to evaluate the risk factors related to the trigeminocardiac reflex (TCR) in patients undergoing Onyx embolization and under general anesthesia. It was shown that: (1) most patients were diagnosed with arteriovenous malformations; (2) 59 patients developed some degree of TCR; (3) TCR was more related to patients with dural arteriovenous fistula and embolization of the middle meningeal artery; and (4) postoperative adverse events were more likely to occur in the TCR group. The authors conclude that dural arteriovenous fistula (DAVF) and embolization of the middle meningeal artery (MMA) are the risk factors that are independent of TCR during Onyx embolization.

In the fourth original paper by Xiao et al. an “imperfect” end-to-side (ES) anastomosis was performed in rats to simulate different scenarios during bypass procedures. The common iliac artery (CIA) was exposed, and three types of end-to-side anastomosis were executed: proximal CIA to contralateral CIA, distal end of the CIA to the ipsilateral side of the common iliac vein. The authors conclude that practice with these proposed models can offer a good alternative for the improvement of bypass techniques in real operations.

In the fifth original paper, Zhang et al. aimed to evaluate the feasibility and effectivity of microvascular anastomosis using 3D exoscope in an *in vivo* animal study. They compared the use of conventional microscope and 3D exoscope during end-to-end (EE) anastomosis performed in the abdominal aorta on mice. The authors assessed the dissection duration, temporary occlusion duration, total procedure duration, and blood leakage at the sutures. They also examined vascular patency under sodium fluorescein angiography. The conclusions were that conventional microscope and 3D exoscope are comparable outcomes for microvascular anastomosis in terms of duration of the procedure, feasibility, and patency.

In the sixth original article, D’Andrea et al. proposed to validate a simplified, ready-to-use, reusable, and ergonomic bypass simulator. This simulator was evaluated by 12 novice and two expert vascular neurosurgeons. Each group performed eight EE, eight ES, and eight side-to-side (SS) microanastomoses using 2 mm synthetic vessels. The results presented demonstrated that the novice group showed improvement in the time to perform the three types of microanastomoses. In general, the more training was performed on the bypass model, the less time to perform them progressively. Such results suggest that the model proposed is efficient to improve the hand–eye coordination and dexterity in performing the microanastomoses.

The first case report by Wei et al. described the case of a 40-year-old male who was diagnosed with a ruptured fusiform aneurysm in the M1 segment of the middle cerebral artery. The authors chose the use of Tubridge flow diverters combined with coil embolization. The treatment proposed by the authors proved to be a good choice, with the patient presenting good results in the short term, and the angiography performed 2 and 12 months after surgery showing total occlusion of the aneurysm and good recovery of the patient.

The second case report by Liao et al. presented the case of a 59-year-old male, in whom the CT scan demonstrated a large aneurysm in the M1 portion of the left middle cerebral artery and a small aneurysm in the M2 portion. Surgical clipping was chosen. However, postoperatively, the patient was in coma and presented fever and high levels of creatine phosphokinase and creatine phosphokinase isoenzyme, indicating failure in kidney and liver function. Postsurgical rhabdomyolysis was diagnosed. The patient received anti-infective treatment, rehydration, urine alkalinization, preservation of renal and hepatic functions, and hemodialysis, which showed beneficial results.

In the third case report, Xu et al. present the case of a 29-year-old male with paroxysmal involuntary twitching of the left masticatory muscles (hemimasticatory spasm—HMS) accompanied by pain related to Parry–Romberg syndrome (PRS). As treatment, surgical resection of 50% of the cephalic and caudal branches of the motor root of the trigeminal nerve was performed, together with the communicating branch. The patient showed a significant improvement after treatment, with the disappearance of spasms and the absence of pain. Therefore, the authors concluded that partial resection of the trigeminal nerve motor branch presents good benefits in the treatment of HMS secondary to PRS.

The review paper by Nguyen et al. aimed to present the indications and microsurgical techniques, discussing step-by-step the bypass variants with technical pearls for the treatment of adult moyamoya disease (MMD). First, the authors presented the indications of surgery for patients with MMD. Second, different bypass techniques, starting with indirect bypass, passing through direct bypass, and ending with combination of these techniques. The authors end this review by presenting a case of an MMD patient treated with direct bypass double barrel/2D2R, and tips on what to do and what to avoid during an MMD surgery.

Liu et al. presented a mini-review of the literature on renovascular hypertension associated with MMD. Epidemiological data have shown that renal artery stenosis may be more frequent in chronic MMD disease, especially in children and Asian individuals. Furthermore, there seems to be a participation of mutations in the RNF213 gene. The authors also present a review of clinical studies showing the morphological and clinical characteristics of this type of renal artery stenosis associated with MMD, ending this mini-review with treatments that can be intravascular intervention with balloon angioplasty, surgical treatment by autotransplantation, and drug treatment.

In summary, this Research Topic for Frontiers in Surgery: *Vascular Neurosurgery and Microneuroanatomy* presented an overview of the current advances in the aspect of cerebrovascular diseases and treatment research and ongoing research directions. The papers discussed above have emphasized the importance of this area of research and highlighted additional works needed.

